# Fatty Liver Disease in Australia: A Narrative Review on the Epidemiology, Natural History, Prognostication and Management in People With Metabolic Dysfunction

**DOI:** 10.5694/mja2.70195

**Published:** 2026-05-10

**Authors:** Karl Vaz, Daniel Clayton‐Chubb, William W. Kemp, Stuart K. Roberts, Ammar Majeed

**Affiliations:** ^1^ Austin Health Melbourne Victoria Australia; ^2^ University of Melbourne Melbourne Victoria Australia; ^3^ Monash University Melbourne Victoria Australia; ^4^ Alfred Health Melbourne Victoria Australia

**Keywords:** epidemiology, morbidity, mortality, public health

## Abstract

Metabolic (dysfunction)‐associated fatty liver disease (MAFLD) or metabolic (dysfunction)‐associated steatotic liver disease (MASLD) is the most common and fastest growing cause of chronic liver disease worldwide. There has been a substantial increase in the epidemiological research regarding MASLD/MAFLD originating from Australia since 2020. This narrative review summarises these pivotal epidemiological studies investigating the disease prevalence, natural history, prognostication and management of this condition. The Australian literature demonstrates the prevalence to be between one‐third and two‐fifths of adults affected, depending on nomenclature, with a heightened risk of cardiovascular disease irrespective of terminology. Current local data support guideline‐based disease staging with non‐invasive tests of fibrosis and the management continues to centre on diet and lifestyle interventions, with directed therapy on the horizon.

## 1 Introduction

Over the past half‐century, non‐alcoholic fatty liver disease (NAFLD)—or its revised term metabolic (dysfunction)‐associated fatty liver disease (MAFLD)—has become the principal cause of chronic liver disease, with a contemporary global meta‐analysis revealing that over one‐third of adults are living with this condition [1]. The prevalence has ballooned in parallel with the obesity and diabetes epidemics [2], with these diseases intimately linked through the shared pathogenic hallmark of insulin resistance [3, 4]. Importantly, each contributes to the broader cardiometabolic syndrome [5], which accounts for high morbidity and premature mortality across many organ systems [3, 6].

Before 2020, there were few studies from Australia—or indeed Oceania—related to the epidemiology and clinical burden of NAFLD/MAFLD despite the high prevalence of obesity in the region [7]. An earlier Markov model imputing primarily international data forecast the incidence and prevalence of NAFLD to rise in Australia over this decade, with an associated substantial economic cost [8]. More recently, several landmark studies have been published in the field addressing this knowledge gap and providing vital local data to inform our national public health discourse. This has culminated in the development of the first multi‐societal clinical practice guideline for the screening, surveillance and monitoring of MAFLD for primary care practitioners in Australia, which was published in the *Medical Journal of Australia* in 2025 [9].

**FIGURE 1:**
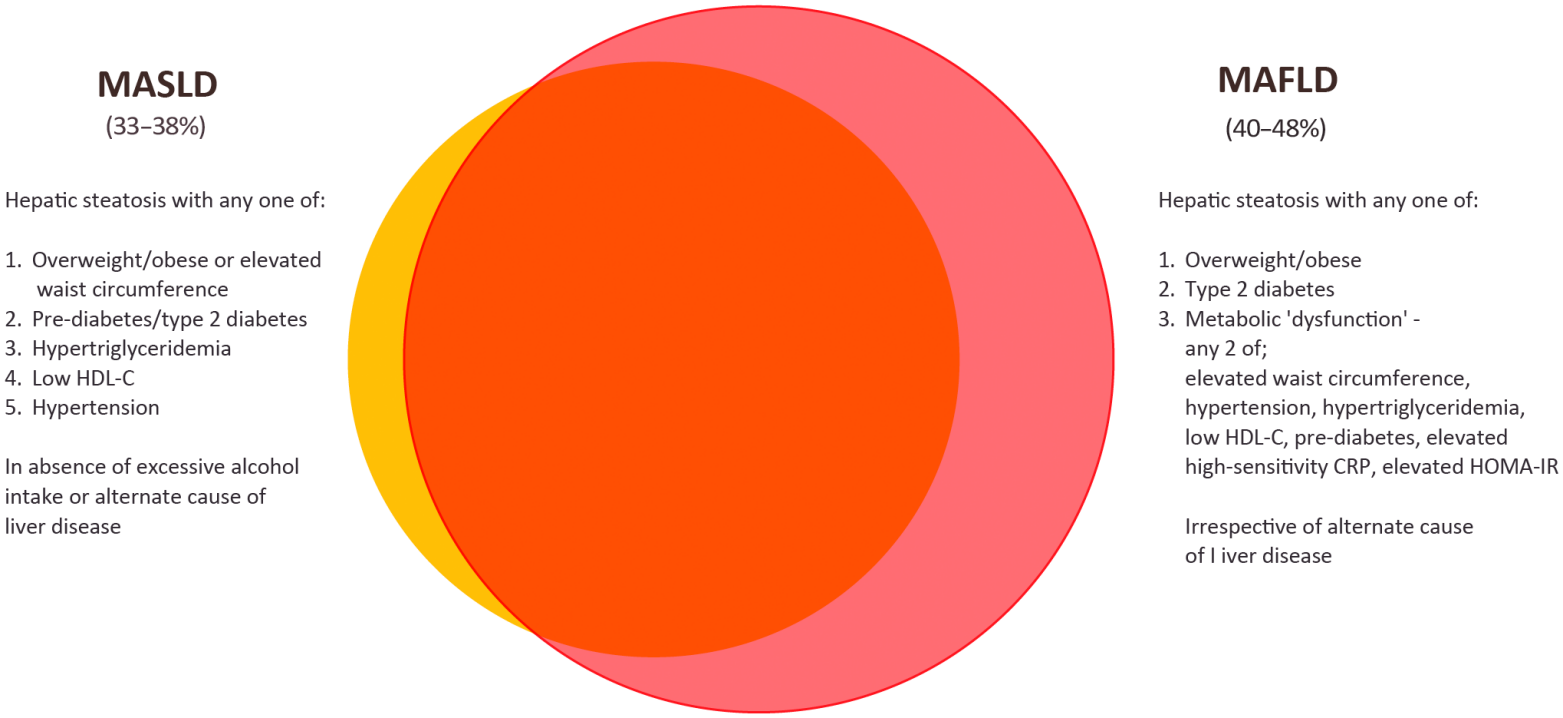
Prevalence and diagnostic criteria of metabolic (dysfunction)‐associated fatty liver disease (MAFLD; red) and metabolic (dysfunction)‐associated steatotic liver disease (MASLD; orange) in Australia. CRP, C‐reactive protein; HDL‐C, high‐density lipoprotein cholesterol; HOMA‐IR, homeostatic model assessment for insulin resistance.

Furthermore, the nomenclature has undergone transformation since 2020, primarily to reflect the underlying disease pathophysiology related to metabolic dysfunction [10]. The two updated terms—MAFLD and metabolic (dysfunction)‐associated steatotic liver disease (MASLD)—have similar diagnostic criteria based on detection of hepatic steatosis in the presence of metabolic dysfunction, with the principal point of difference pertaining to the need to rule out alternate causes of liver disease in MASLD but not MAFLD (Figure 1) [11, 12]. As such, MASLD can be considered akin to NAFLD [13], whereas MAFLD is a broader diagnosis that may also confer a more hazardous prognosis due to potentially multiple liver pathologies co‐existing [14]. To avoid confusion in this article, NAFLD and MASLD will be considered interchangeably and will herein be referred to as MASLD.

Given the above, this is an opportune time to synthesise the local studies investigating the epidemiology, natural history, prognostication and management of MASLD/MAFLD in Australia. This narrative review aims to concisely present the evidence to date, with a focus on the adult literature, and considers areas for future research focus.

## 2 Methodology

We performed a literature search of PubMed for articles related to NAFLD, MAFLD and/or MASLD on Australian cohorts and pertaining to epidemiology, clinical outcomes and management, published up until September 2025. References of relevant studies were additionally checked for applicability. Paediatric and adolescent (i.e., < 18‐year‐olds) cohorts were excluded. Alcohol‐associated fatty liver disease is not considered in this review. Sex is categorised as male/female, according to biological designation at birth.

## 3 Epidemiology

The crude prevalence of MASLD/MAFLD from Australian adult cohort studies, including key demographic details, is summarised in Table 1. Earlier studies investigating the prevalence of MASLD in Australia adopted elevated alanine aminotransferase (ALT) as a surrogate measure for MASLD [15, 16]. Adams and colleagues found the prevalence to be 9% in 1994–1995 [15], while Mahady and colleagues found it to be 11.2% in 2011–2012 [16]. However, elevated ALT as a diagnostic method for hepatic steatosis is both poorly sensitive and non‐specific [24], thus considered insufficient for modern epidemiological studies. Despite imaging being the ideal surrogate for biopsy‐proven hepatic steatosis, the community‐based Raine Study is the only Australian study on a general cohort to utilise this in investigating MASLD prevalence, noted to be 17% in 2016–2018 among 588 participants aged 27 years who underwent magnetic resonance imaging [17].

**TABLE 1:** Summary of cohort studies investigating metabolic (dysfunction)‐associated fatty liver disease or metabolic (dysfunction)‐associated steatotic liver disease prevalence in Australia.

Author	Cohort (year)	Sample size	Demographics^a^	Diagnostic method	Prevalence^b^
Adams et al. [15]	Busselton Health Study (1994–1995)	2610	Age, 51.8 years (SD, 15.3 years) Male/female, 51%/49% BMI, 26.2 (SD, 4.0) Overweight/obese, 41% WC, 87.3 cm (SD, 12.4 cm) Elevated WC, 22%	Elevated ALT (≥ 40 U/L)	9% MASLD
Mahady et al. [16]	Australian Health Survey (2011–2012)	9447	Male/female, 50%/50% Elevated WC, 62% Diabetes, 4.6%	Elevated ALT (≥ 40 U/L males, ≥ 30 U/L females)	11.2% MASLD
Tashkent et al. [17]	The Raine Study Generation 2 (2016–2018)	588	Age, 27 years Male/female, 48%/52% BMI, 24 (IQR, 25–50)	Magnetic resonance imaging volumetric liver fat fraction > 3.55%	17% MASLD
Roberts et al. [18]	Crossroads II (2016–2018)	705	Age, 59.1 years (SD, 16.1 years) Male/female, 44%/56% BMI, 29.7 (SD, 19.6) Overweight/obese, 74.9% WC, 99 cm (SD, 15 cm) Elevated WC, 58.4% Diabetes, 12%	FLI ≥ 60	38.9% MASLD
Kemp et al. [19]	Crossroads II (2016–2018)	722	Age, 59.3 years (SD, 16.0 years) Male/female, 45%/55% BMI, 27.8 (SD, 7.3) Overweight/obese, 75.2% WC, 98.5 cm (SD, 18.9 cm) Elevated WC, 58.5% Diabetes, 9.7%	FLI ≥ 60	47.2% MAFLD
Farrell et al. [20]	AusDiab 3rd Survey (2012)	4747	Age, 60 years (IQR, 53–68 years) Male/female, 45%/55% Overweight/obese, 68% Diabetes, 10%	FLI > 60	37% MAFLD
Vaz et al. [21]	Crossroads I and II (2001–2003; 2016–2018)	1040; 704	Crossroads I: ○ Age, 52.8 years (SD, 15.7 years) ○ Male/female, 44%/56% ○ BMI, 27.9 (SD, 5.4) ○ Overweight/obese, 68.2% ○ WC, 94.7 cm (SD, 14.4 cm) ○ Elevated WC, 48.2% ○ Diabetes, 7.3% Crossroads II, as above	FLI ≥ 60	32.7% → 38.8% MASLD
Clayton‐Chubb et al. [22, 23]	ASPREE (2010–2014)	9847	Age, 75.1 years (SD, 4.3 years) Male/female, 45%/55% BMI, 27.8 (SD, 4.4) Overweight/obese, 72.9% WC, 96.1 cm (SD, 12.3 cm) Elevated WC, 54.6% Diabetes, 9.2%	FLI ≥ 60	33% MASLD; 38% MAFLD

Abbreviations: ALT, alanine aminotransferase; ASPREE, Aspirin in Reducing Events in the Elderly study; AusDiab, Australian Diabetes, Obesity and Lifestyle study; BMI, body mass index; FLI, Fatty Liver Index; IQR, interquartile range; MAFLD, metabolic (dysfunction)‐associated fatty liver disease; MASLD, metabolic (dysfunction)‐associated steatotic liver disease; SD, standard deviation; WC, waist circumference.

^a^ Central value presented as mean (SD) or median (IQR).

^b^ Data presented as crude prevalence.

The Fatty Liver Index (FLI) is a readily available and validated score used to diagnose hepatic steatosis [25] and is endorsed by major societal guidelines for use in epidemiological studies [26, 27]. Since 2021, several studies have investigated the point prevalence of FLI‐determined MASLD/MAFLD from the population‐based Crossroads and Australian Diabetes, Obesity and Lifestyle (AusDiab) studies, each of which contributes to the Australian and New Zealand Diabetes and Cancer Collaborative [28, 29]. Roberts et al. established the age‐ and sex‐standardised MASLD prevalence to be 35.7% in Crossroads II (CR‐2) between 2016 and 2018 in a predominantly white cohort (93%) [18]. Considering people with overweight/obesity, diabetes and the metabolic syndrome, MASLD prevalence was 51%, 61% and 70% [18], respectively, highlighting metabolic dysfunction as intrinsically linked to the disease. The crude MAFLD prevalence from CR‐2 was 47.2% [19], with the difference in MASLD and MAFLD prevalence due to the more inclusive MAFLD diagnostic criteria (Figure 1) [11]. Farrell and colleagues found the MAFLD prevalence from the AusDiab study to be 37% [20]. Despite the geographically wider population coverage in enrolment compared with Crossroads, it had comparable baseline demographic details (Table 1) [20].

The analysis by Vaz et al. from both Crossroads studies (2001–2003 and 2016–2018) identified a significant increase in the age‐ and sex‐standardised MASLD prevalence (32.4% [95% confidence interval (CI), 29.5%–35.4%] to 35.4% [95% CI, 31.3%–39.5%]; *p* < 0.01) [21]. Furthermore, there was a greater prevalence rise in females than males and the increase occurred in parallel to a rising prevalence of central and generalised obesity and frequency of takeaway food consumption [21]. Although there was a higher prevalence of MASLD in rural versus regional centres in this study, no geographic difference (urban vs. regional vs. rural) was established from the AusDiab study [20, 21].

Clayton‐Chubb and colleagues determined the MASLD prevalence in older age Australians—a previously understudied group across the world [22]. In a cross‐sectional analysis of ~10,000 Australian participants enrolled into the Aspirin in Reducing Events in the Elderly (ASPREE) randomised controlled trial (RCT) between 2010 and 2014 with evaluable laboratory data, the FLI‐determined MASLD prevalence in people aged ≥ 70 years was 33.0%, with a progressive decline in prevalence from 70–72 years to ≥ 85 years [22]. MAFLD prevalence from this cohort was 38% [23]. Notably, a relevant exclusion to enrolment into ASPREE was any prior history of clinically evident cardiovascular or cerebrovascular disease, representing a healthier cohort than what can be expected in the general population for this age strata [22].

Another understudied area of MASLD/MAFLD is the impact of dual aetiologies of liver disease (e.g., hepatosteatosis and viral hepatitis). Two local studies have estimated the prevalence of co‐existent MASLD to be 14%–18% among those with chronic hepatitis B [30, 31], but we lack Australian data on concurrent hepatitis C virus or other causes of chronic liver disease and MASLD.

## 4 Natural History

For people living with MASLD/MAFLD, clinicians and policymakers alike, natural history studies are critical to provide information on prognosis, focus selection of high‐risk subgroups to target for disease‐modifying interventions and to act as a reference group against which public health interventions can be assessed.

### 4.1 Adverse Liver‐Related Outcomes

The foremost liver‐related sequelae pertain to the development of decompensated liver disease (ascites, encephalopathy, variceal bleeding), preceded by cirrhosis, and hepatocellular carcinoma, each of which are indications for liver transplantation. O'Beirne and colleagues investigated the liver disease progression of MASLD through a retrospective cohort study utilising linked Queensland health datasets [32]. In this analysis of over 10,000 admissions between 2007 and 2019, with a median follow‐up of 4.6 years, 4.4% of people with MASLD progressed to decompensated cirrhosis according to the International Classification of Diseases, tenth revision, Australian modification (ICD‐10‐AM) coding [32]. Diabetes was highlighted as a key risk factor for progression to decompensated liver disease, conferring a threefold increased risk in people without cirrhosis and a 14‐fold increased risk occurring together with cirrhosis [32]. Although diabetes was more prevalent among First Nations peoples with MASLD in another Queensland study, there was no association between Indigenous status and progression to decompensated cirrhosis [33].

In a linkage study using the Crossroads I (CR‐1) population‐based cohort, with a median 19.7 years' follow‐up, Vaz and colleagues demonstrated metabolic syndrome risk factors to have an additive risk on incident decompensated liver disease in both MASLD and MAFLD [34]. Less than 0.5% of MASLD/MAFLD participants developed primary liver cancer from this community‐based cohort. Another linkage study from Western Australia (WA) investigated the 5‐year cumulative risk of decompensated cirrhosis (4.7%; 95% CI, 3.5%–6.2%), hepatocellular carcinoma (1.4%; 95% CI, 0.9%–2.3%), liver transplantation (0.95%; 95% CI, 0.56%–1.61%) and liver‐related death (2.8%; 95% CI, 2.0%–3.9%) in 1597 people with MASLD, and determined this to be lower for most adverse events compared with other aetiologies of liver disease (except chronic hepatitis B) [35]. Finally, studies have revealed MASLD‐associated hepatocellular carcinoma and liver transplant for MASLD to have significantly risen over the past three decades in Australia [36, 37].

### 4.2 Extrahepatic Morbidity and Mortality

A consistent finding from the international literature is extrahepatic morbidity and mortality, especially related to cardiovascular disease (CVD) and extrahepatic cancer, occurring more frequently in people with MASLD/MAFLD than progression to decompensated cirrhosis [38, 39, 40]. In another CR‐1 analysis, both MASLD and MAFLD were found to be independently associated with a 1.5‐fold increased risk of 3‐point major adverse cardiovascular events (MACE) after controlling for established cardiometabolic risk factors, but neither was independently associated with atrial fibrillation [41]. In contrast, the ASPREE analysis found MASLD was independently associated with a 1.5‐fold increased risk of atrial fibrillation over a median 4.4 years' follow‐up [42]. Furthermore, in this older cohort, MASLD was independently associated with persistent physical disability, but, interestingly, it was associated with a reduced risk of dementia [43].

Thus, the present Australian literature supports the notion that there are bidirectional links between metabolic syndrome risk factors upon MASLD/MAFLD prevalence and severity, and conversely, MASLD/MAFLD on adverse metabolic syndrome sequelae. Despite the increased morbidity, no increase in CVD‐related or all‐cause mortality has been detected in individuals with MASLD/MAFLD in either the general population of CR‐1 nor the ASPREE trial cohorts [34, 42]. This is consistent with literature abroad [40] and reflects the heterogeneity in disease severity/stage occurring within the umbrella of MASLD/MAFLD, highlighting the importance of patient‐focused risk stratification.

## 5 Prognostication

The literature unequivocally reveals the stage of liver fibrosis to be the foremost predictor for adverse liver outcome (i.e., decompensated disease or death) in MASLD/MAFLD [38, 44, 45, 46]. Given several issues related to liver biopsy solely for determining fibrosis stage, including its invasive nature, accessibility, sampling error, cost and intra‐ and/or inter‐rater reporting variability [47], surrogate measures or non‐invasive tests have been developed for liver fibrosis staging. These non‐invasive tests can be broadly categorised into blood‐based markers, radiological markers or composites of both, and as either direct or indirect markers of liver fibrosis. These tests signpost the setting and manner in which people with MASLD/MAFLD should undertake surveillance and management [9].

### 5.1 Determining Stage of Fibrosis

Patel and colleagues conducted a prospective study in a single tertiary referral centre in Queensland, enrolling 252 people with MASLD between 2015 and 2017 to undertake comprehensive liver fibrosis staging, including the Fibrosis‐4 Index (FIB‐4), the NAFLD Fibrosis Score (NFS), the Enhanced Liver Fibrosis (ELF; Siemens Healthineers) test, vibration‐controlled transient elastography (VCTE; i.e., FibroScan [Echosens]) and a subset (19%) with liver biopsy [48]. This study was enriched with a greater proportion of advanced fibrosis/cirrhosis (15%) than that expected from a community‐based cohort, given participants were enrolled from diabetes clinics or deemed to have high‐risk features in primary care [48]. Given this high prevalence, the sensitivity of FIB‐4 and NFS for ruling out significant fibrosis based on ELF or VCTE was low when used singularly, but 90% when both non‐invasive tests were low. Similarly, concordant elevation in ELF (≥ 9.8) and VCTE (≥ 8.2 kPa) had a 96% positive predictive value for predicting biopsy‐proven advanced fibrosis/cirrhosis [48].

In the largest biopsy‐confirmed MASLD study in a single academic centre from WA conducted on 271 people between 2004 and 2018, Bertot and colleagues found the negative predictive value of FIB‐4 for ruling out advanced fibrosis to be ~86% and greater than other first‐line non‐invasive tests [49]. As above, this was a cohort enriched with advanced fibrosis (31%). The same group demonstrated that the presence of diabetes attenuated the discriminatory capacity of non‐invasive tests in predicting advanced fibrosis/cirrhosis, with Hepascore (PathWest) the only surrogate test maintaining an area under the receiver operating characteristic curve ≥ 0.80 in individuals with diabetes [50]. This is consistent with international literature demonstrating that first‐line non‐proprietary non‐invasive tests have substandard performance in people with diabetes [51].

Current guidelines recommending using the FIB‐4 to rule out advanced fibrosis are supported by the above data [9]. The guidelines suggest referral for hepatology opinion or proprietary non‐invasive test (VCTE, ELF, Hepascore) when the FIB‐4 is > 1.3 [9], but none of these second‐line tests are covered by Medicare at present and, consequently, cost is a barrier to the widespread adoption of these more accurate non‐invasive tests.

### 5.2 Predicting Clinical Outcomes

Several studies have validated the use of liver fibrosis non‐invasive tests in predicting clinical outcomes. In a follow‐up from the aforementioned Queensland study, with a median 4.2 years' follow‐up time, elevated baseline liver stiffness measurement on VCTE was the best predictor of liver‐related event among the non‐invasive tests [52]. Similarly, Hepascore outperformed first‐line non‐invasive tests in predicting a liver‐related event in a multicentre cohort of 872 MAFLD participants in WA [53]. One study developed a novel score—the NAFLD Outcomes Score—including readily available clinical and biochemical variables to predict liver‐related events, outperforming repurposed liver fibrosis non‐invasive tests in both the derivation (from WA) and validation (international) cohorts [54].

In a large retrospective multicentre study from Victoria between 2008 and 2019, enrolling > 6000 people with MASLD who had undertaken VCTE and linked to ICD‐10‐coded clinical outcomes, elevated liver stiffness measurement was independently associated with all‐cause mortality over 22,653 person‐years follow‐up time, with a stepwise increase in mortality for every 5 kPa rise in liver stiffness measurement [55]. Vaz and colleagues demonstrated multiple non‐invasive tests comprised of conventional blood tests to all have moderate‐to‐excellent predictive performance for all‐cause, CVD‐related and extrahepatic cancer‐related mortality in individuals with MASLD and MAFLD from the community‐based CR‐1 cohort [56]. Furthermore, MASLD or MAFLD nomenclature did not influence the proportion of participants with indeterminate or high‐risk FIB‐4 or NFS (i.e., those recommended for hepatology referral) [57]. Analysis from ASPREE revealed several serum surrogate markers for hepatic steatosis to be associated with MACE, atrial fibrillation and physical disability [58], and that low ALT levels were associated with a heightened risk of all‐cause mortality [59].

An alternative approach to risk‐stratification is the use of genetic testing. Multiple variants in genes related to lipid handling have been associated with progressive MASLD/MAFLD. When assessing their impact on outcomes in older persons, none conferred an increased risk for MACE nor all‐cause mortality in the ASPREE cohort [60], perhaps due to survival bias and the a priori selection of individuals without prior clinically evident atherosclerotic CVD.

Taken together, the literature from Australia not only endorses the use of non‐invasive tests in individuals diagnosed with MAFLD as per current clinical guidance to stage liver fibrosis [9], but an elevated score (i.e., FIB‐4 > 1.3) should indicate to practitioners the risk of adverse hepatic and extrahepatic outcomes. Although this could signal the need for managing other metabolic syndrome risk factors to reduce the risk of CVD and ensure diligence in age‐ and sex‐appropriate malignancy surveillance, future research is required to validate the efficacy of utilising non‐invasive tests in this manner.

## 6 Management

### 6.1 Diet and Lifestyle

For decades, the cornerstone of MASLD/MAFLD management has been through diet and lifestyle modification and assessing for and treating metabolic syndrome risk factors to both prevent liver disease progression and reduce the risk of incident extrahepatic sequelae, particularly CVD [9, 61, 62]. Weight loss has been promoted, primarily through targeting an energy deficit with hypocaloric diet achieved through various means, as thresholds of 7% and 10% weight reduction have led to metabolic (dysfunction)‐associated steatohepatitis (MASH) resolution and fibrosis regression in clinical trials [63]. However, this is both challenging to achieve and maintain at an individual and population level in the absence of anti‐obesity medications and/or bariatric surgery.

In an Australian pilot study (*n* = 12), adult participants with biopsy‐proven MASLD were randomised to either a Mediterranean diet or a low‐fat‐high carbohydrate diet (LF‐HCD) for 6 weeks, before switching arms following a washout period [64]. The Mediterranean diet was associated with a greater reduction in quantifiable hepatic steatosis and improvements in markers of insulin sensitivity compared with the LF‐HCD [64]. However, in two larger RCTs (*n* = 42 and *n* = 48) from Australia assessing the efficacy of the Mediterranean diet versus the low‐fat diet, neither study revealed any difference in hepatic fat content nor insulin resistance between the two diets over a 12‐week period; whereas in the study by George and colleagues, both diet types led to reduction in visceral fat [65, 66]. In post hoc analysis from ASPREE, greater adherence to a Mediterranean diet pattern was associated with reduced prevalent MASLD [67].

### 6.2 Emerging Pharmacotherapy

There is a burgeoning pipeline of pharmacotherapies being trialled for MASH worldwide. Semaglutide (a glucagon‐like peptide 1 agonist [GLP‐1]) was shown in a seminal phase 3 RCT to improve both histological steatohepatitis and induce fibrosis regression in non‐cirrhotic disease [68]. It has subsequently been licensed in the United States (US) [69], but its efficacy in established MASH‐related cirrhosis remains unknown, with phase 3 trials currently underway. Multiple other GLP‐1‐based therapies alone or in combination with other incretin‐mimetics/entero‐endocrine agonists are currently being assessed for use in fibrotic MASH [70]. Semaglutide has been approved by the Therapeutic Goods Administration in 2026 to treat non‐cirrhotic MASH in Australia, the first medication to be approved to treat this condition. These agents offer the tantalising prospect of managing multiple sequelae of metabolic syndrome with a single therapy.

Mechanistically novel MASH‐specific drugs are also in various stages of development and trial. Resmetirom—a first‐in‐class thyroid hormone receptor β‐agonist—was the first licensed MASH pharmacotherapy in the US [71], based on the pivotal MAESTRO‐NASH phase 3 RCT [72]. Another therapeutic class of interest is the fibroblast growth factor‐21 (FGF‐21) agonists [73], which have reported promising results from early trials [74, 75], even demonstrating cirrhosis regression after 96 weeks of therapy [76]; phase 3 trials are underway in Australia. Combination regimens are also under development, with a small proof‐of‐concept study providing preliminary evidence of safety and efficacy when combining various metabolic‐targeting agents [77]. This is relevant given that there remain many non‐responders to therapy in the registration trials noted above.

It remains to be seen if any of these agents for these indications will be approved by the Therapeutic Goods Administration and the Pharmaceutical Benefits Advisory Committee, as there are uncertainties on how best to select patients for treatment with currently available non‐invasive tests, monitoring for efficacy/futility on treatment, duration of use and impact on hard clinical endpoints. Notably, though, the Australian Diabetes Society clinical care guidelines for managing MAFLD in individuals with diabetes recommend the use of anti‐obesity pharmacotherapy, with a preference for incretin‐mimetics, in those with these co‐existent conditions or MAFLD in the presence of obesity [78].

## 7 Research Priorities in Australia

Although the epidemiological data for MASLD/MAFLD in adults in Australia have matured substantially over the past 5 years, there remain several unanswered questions. Future local research efforts should focus on prospective studies through different care settings (primary to tertiary) and in people who are under‐represented (e.g., Aboriginal and Torres Strait Islander people [79]), evaluate the impact of changing metabolic syndrome parameters and anthropometry on clinical outcomes, the establishment and evaluation of novel models of care, the development of MASLD/MAFLD‐specific risk assessment tools for predicting clinical outcomes, and developing material to educate the public (Table 2).

**TABLE 2:** Knowledge gaps and future research priorities, including select active studies in Australia.

Knowledge gaps	Research priorities
Epidemiology and natural history	Evaluation of cohorts with repeat data collected longitudinally (such as the Busselton or AusDiab cohorts) permitting evaluation of how change in anthropometry, biochemistry and clinical features over time influence hepatic and extrahepatic outcomes. Linkage of established datasets to MBS/PBS data to allow analysis of the impact of prescription medication (e.g., aspirin, statins and anti‐diabetes medication, in particular incretin mimetics) on clinical outcome. Establish the risk of extrahepatic cancer in Australians with MASLD/MAFLD and impact of liver disease on the risk of malignancy beyond known risk factors. Understand the impact of MASLD/MAFLD on Aboriginal and Torres Strait Islander people, such as through the use of the DRUID study, and how this differs from the non‐Indigenous population. Evaluate the impact of MASLD/MAFLD across various stages of disease (non‐fibrotic, fibrotic, cirrhosis) on quality of life through the use of validated questionnaires.
Prognostication	Developing accurate clinical predictor tools to identify people with at‐risk (i.e., fibrotic) MASH in primary care (e.g., study ACTRN12623001183673). Developing hepatocellular carcinoma risk‐predictor tools to determine which people with MASLD/MAFLD without cirrhosis will most benefit from routine surveillance. Developing CVD risk‐predictor tools incorporating MASLD/MAFLD and evaluating their discriminatory capacity compared with the current standard of care (Australian CVD Risk Calculator; https://www.cvdcheck.org.au/calculator) and validating them in large community‐based cohorts. NAFLD‐RRR study (ACTRN12621000330842)–determining the efficacy of FibroScan (Echosens) for detecting MASLD in a type 2 diabetes clinic.
Management	Randomised trials of novel models of service delivery (e.g., nurse‐led MASLD/MAFLD clinics, dedicated MASLD/MAFLD clinics, two‐tier fibrosis staging within primary care) to assess the safety, efficacy and cost‐effectiveness of these models of care. TNT study (ACTRN12619000701123)—double‐blind, placebo‐controlled trial investigating the efficacy of testosterone replacement in males with MASLD and low serum testosterone on hepatic steatosis. Efficacy of MASH‐specific therapies on hard clinical endpoints (preventing progression of liver disease, liver decompensation, need for liver transplant and liver‐related death).

Abbreviations: ACTRN, trial registration number on Australia and New Zealand Clinical Trials Registry; AusDiab, Australian Diabetes, Obesity and Lifestyle study; CVD, cardiovascular disease; DRUID study, Darwin Region Urban Indigenous Diabetes study; MAFLD, metabolic (dysfunction)‐associated fatty liver disease; MASH, metabolic (dysfunction)‐associated steatohepatitis; MASLD, metabolic (dysfunction)‐associated steatotic liver disease; MBS, Medicare Benefits Schedule; NAFLD‐RRR, Identifying non‐alcoholic fatty liver disease fibrosis risk in type 2 diabetes patients: implementing the right care, in the right place, at the right time study; PBS, Pharmaceutical Benefits Scheme; TNT, Testosterone therapy for the treatment of non‐alcoholic fatty liver disease study.

These endeavours will help guide all those involved in the care of people with MASLD/MAFLD in Australia (i.e., general practitioners, hepatologists, clinicians specialising in cardiometabolic medicine, nurses, dieticians, hospital administrators, guideline developers and policymakers) to finesse existing best‐practice guidelines aimed at concentrating limited resources to provide the highest yield for managing a condition that is highly prevalent, but for many may be clinically quiescent.

## 8 Conclusions

There are now robust data on the epidemiology, natural history and prognostication of MASLD/MAFLD in Australia. The prevalence of MASLD is about 35%, whereas the prevalence of MAFLD is closer to 40% due to its more inclusive diagnosis. For most people with MASLD/MAFLD, the morbidity and mortality burden is predominantly due to CVD and extrahepatic cancer, rather than adverse liver‐related outcomes. Diabetes is a substantial risk for progressive liver disease and liver‐related morbidity/mortality in people with and without cirrhosis. Several readily available surrogate measures of liver fibrosis not only stage the degree of liver fibrosis—the most important risk factor for deleterious liver‐related outcomes—but also prognosticate the risk of extrahepatic morbidity and mortality. Although the management of MASH is transforming from behavioural alterations alone with the advent of pharmacotherapy, the use of these in Australia remains limited due to regulatory restrictions.

## Author Contributions

**Karl Vaz:** conceptualisation, methodology, data curation, writing (original draft), writing (review and editing). **Daniel Clayton‐Chubb:** conceptualisation, methodology, data curation, writing (original draft), writing (review and editing). **William W. Kemp:** methodology, writing (review and editing). **Stuart K. Roberts:** methodology, writing (review and editing). **Ammar Majeed:** conceptualisation, methodology, writing (review and editing).

## Funding

The authors have nothing to report.

## Disclosure

Not commissioned; externally peer reviewed.

## Conflicts of Interest

The authors declare no conflicts of interest.

## Data Availability

This article includes no original data.
